# Functional Outcome and Safety of Endoscopic Treatment Options for Benign Prostatic Obstruction (BPO) in Patients ≥ 75 Years of Age

**DOI:** 10.3390/jcm13061561

**Published:** 2024-03-08

**Authors:** Susanne Deininger, Anna Maria Dieplinger, Wanda Lauth, Lukas Lusuardi, Peter Törzsök, David Oswald, Maximilian Pallauf, Christian Eiben, Julia Peters, Eva Erne, Quirin Zangl, Christian Deininger, Christian Ramesmayer

**Affiliations:** 1 Department of Urology and Andrology, Salzburg University Hospital, Paracelsus Medical University, 5020 Salzburg, Austria; s.deininger@salk.at (S.D.); l.lusuardi@salk.at (L.L.); torzsok.peter@gmail.com (P.T.); d.oswald@salk.at (D.O.); m.pallauf@salk.at (M.P.); c.eiben@salk.at (C.E.); j.peters@salk.at (J.P.); 2 Institute for Nursing Science and Practice, Paracelsus Medical University, 5020 Salzburg, Austria; annamaria.dieplinger@ooeg.at; 3 Team Biostatistics and Big Medical Data, IDA Lab Salzburg, Paracelsus Medical University Salzburg, Strubergasse 16, 5020 Salzburg, Austria; wanda.lauth@pmu.ac.at; 4 Faculty of Health and Sport Sciences, Széchenyi István University, 9026 Györ, Hungary; 5 Department of Urology, Eberhard Karls University, 72076 Tübingen, Germany; eva.erne@med.uni-tuebingen.de; 6 Department of Neuroanesthesia, Christian Doppler Hospital, Paracelsus Medical University, 5020 Salzburg, Austria; q.zangl@salk.at; 7 Department of Orthopedics and Traumatology, Salzburg University Hospital, Paracelsus Medical University, 5020 Salzburg, Austria; c.deininger@salk.at; 8 Institute of Tendon and Bone Regeneration, Paracelsus Medical University, Spinal Cord Injury & Tissue Regeneration Center Salzburg, 5020 Salzburg, Austria

**Keywords:** benign prostatic obstruction, BPH, transurethral, septuagenarian, TURP, HoLEP

## Abstract

**Background:** The selection of suitable patients for the surgical treatment of benign prostatic obstruction (BPO) is a challenge in persons ≥75 years of age. **Methods**: After a systematic literature search of PubMed, 22 articles were included in this review. Clinical and functional parameters were evaluated statistically. **Results**: The mean age of the patients was ≥79 years. The mean duration of postoperative catheterization ranged between 2 (d) (ThuLEP, thulium laser enucleation of the prostate) and 4.4 days (TURP, transurethral resection of the prostate). Complication rates ranged between 6% (HoLAP, holmium laser ablation of the prostate) and 34% (PVP, photoselective vaporization of the prostate); the maximum rate of severe complications was 4% (TURP). The mean postoperative maximal urinary flow (Qmax) in mL/sec. ranged between 12.9 mL/sec. (HoLAP) and 19.8 mL/sec (Hol-TUIP, holmium laser transurethral incision of the prostate). The mean quality of life (QoL) score fell from 4.7 ± 0.9 to 1.8 ± 0.7 (HoLEP), from 4.1 ± 0.4 to 1.9 ± 0.8 (PVP), from 5.1 ± 0.2 to 2.1 ± 0.2 (TURP), and from 4 to 1 (ThuVEP, thulium laser vapoenucleation of the prostate). Pearson’s correlation coefficient (r) revealed a positive linear correlation between age and inferior functional outcome (higher postoperative International Prostate Symptom Score (IPSS) [r = 0.4175]), higher overall complication rates (r = 0.5432), and blood transfusions (r = 0.4474) across all surgical techniques. **Conclusions**: This meta-analysis provides the summary estimates for perioperative and postoperative functional outcome and safety of endoscopic treatment options for BPO in patients ≥ 75 years of age. Of particular importance is that all surgical techniques significantly improve the postoperative quality of life of patients in this age group compared to their preoperative quality of life.

## 1. Introduction

Benign prostatic obstruction (BPO) is among the most commonly encountered conditions in urologic care [1]. According to the Global Burden of Disease Study 2019, the age-standardized prevalence of BPO was 1.620/100,000 in Germany and 2.250/100,000 in Western Europe [2]. Age is an independent risk factor for its prevalence and severity [3]. Between the ages of 40 and 80 years, the mean prostate volume increases continuously from a median of 21.4 to a median of 34.0–38 cubic centimeters (cc), while the maximum urinary flow Qmax decreases continuously from a median of 20.5–22.1 to a median of 13.0–13.7 mL/s. Likewise, subjective complaints increase consistently [4,5,6]. Old and very old patients (VOPs) experience BPO in large numbers; the age-specific prevalence of benign prostatic hyperplasia (BPH) confirmed in anatomical studies is as high as 80% in nonagenarians [7]. And VOPs are a difficult patient population in terms of surgical care; postoperative morbidity in prostate surgery is linked to age, polypharmacy, and preoperative catheterization [8].

Low birth rates and longer life expectancy are transforming population demographics in high- and middle-income countries. The oldest defined age group (i.e., those ≥85 years of age) is the fastest expanding section of the population. According to reports, the yearly growth rate of this age group worldwide is 3.8%. One-fifth of the elderly will belong to the oldest old age group by 2050.

This is also expected to cause an absolute and percentage increase in the numbers of BPOs requiring treatment. Various transurethral techniques are currently available for the surgical treatment of BPOs. However, many pivotal studies have been conducted in patients of significantly younger ages. Little is known about perioperative courses and functional outcomes in the VOPs group.

The aim of this work is to systematically review and meta-analyze clinical studies investigating established endoscopic techniques for BPO treatment in patients ≥ 75 years of age.

## 2. Materials and Methods

A part of this report was incorporated in a so-called academic expert thesis for the Master’s program entitled Health Sciences and Leadership at Paracelsus Medical University, Salzburg.

### 2.1. Literature Search

From February to March 2023, the first author performed a literature search on PubMed/Medline to identify existing studies on transurethral surgical procedures performed for the indication of BPO in patients over 75 years of age. Those studies which explicitly included perioperative courses and outcomes in these age groups were included. When multiple follow-up (FU) periods were mentioned, the results for the longest one were reported in the analysis. Studies published over the last 11 years were included (January 2012–March 2023). The following search terms were used in different combinations: 75/85 years, Aquablation® (PROCEPT BioRobotics, 900 Island Drive, 94065 Redwood City, CA, USA), BPO/BPH, elderly, frail, GreenLight XPS™ (Boston Scientific, 300 Boston Scientific Way, 01752 Marlborough, MA, United States), HoLEP, laser, lower urinary tract symptoms (LUTS), octogenarians, photoselective vaporization of the prostate (PVP), prostate, prostatic urethral lift, potassium titanyl phosphate (KTP), Rezum™ (Boston Scientific, 300 Boston Scientific Way, 01752 Marlborough, United States), septuagenarian, transurethral, TURP/transurethral resection, and (very) old. The reference lists of the identified studies were used to locate additional relevant studies within the literature.

### 2.2. Study Selection

The research question was developed using the PICO scheme [9]:

- P (Population): male, age ≥ 75 years, with the indication for surgical therapy for BPO;
- I (Intervention): different endoscopic treatment options for BPO;
- C (Comparison): various;
- O (Outcome): different functional parameters, rate of complications, QoL.

**Figure 1:**
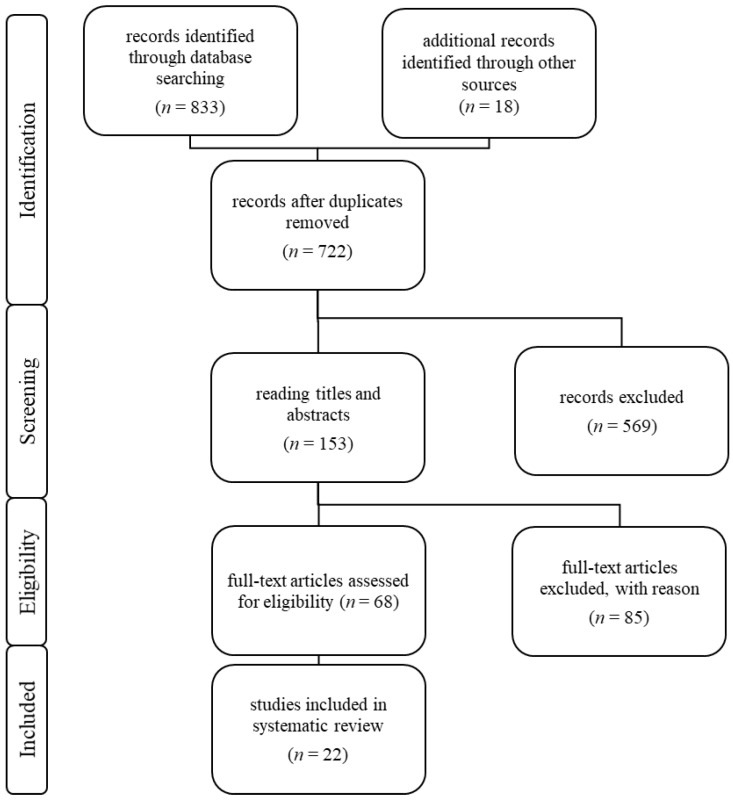
Study selection according to the recommendations of the PRISMA statement.

Studies were selected according to the recommendations of the Preferred Reporting Items for Systematic Review and Meta-analysis Statement (PRISMA) [10]. The study selection process is shown in Figure 1. A total of 833 articles were identified through database research. An additional 18 articles were identified from other sources (compare [11]). Duplicates were omitted. Of the remaining 722 articles, the titles and abstracts of 153 were read. Sixty-eight reports were subjected to a full-text assessment for eligibility. Eighty-five articles were excluded either due to unsuitable content or incomplete mention of the examined parameters. In all 22 articles, a systematic review and meta-analysis were incorporated. Only publications in the English language were considered. Only surgical interventions that comply with the current EAU guidelines were included [12]. The study was registered in the international prospective register of systematic reviews (PROSPERO) with the unique identification number CRD449835 [13].

### 2.3. Cochrane’s Collaboration Tool for Assessing Risk of Bias [14]

Of the selected studies, only one was prospectively randomized [15]. It was examined using Cochrane’s collaboration tool for assessing risk of bias; the result is shown in Table 1.

For all other studies, the risk of bias was considered high.

### 2.4. Data Extraction

Data extraction was performed by the authors. We employed a uniform data table which contained the following: general information about the publication (name, authors, journal, and year of publication), details concerning randomization, baseline characteristics of the patients (number, mean, and standard deviation of age, American Society of Anesthesiologists Classification (ASA), antiplatelet and/or anticoagulation therapy, preoperative and resected prostate volume, preoperative and postoperative post-void residual volume (PVR), preoperative and postoperative prostate-specific antigen (PSA), preoperative and postoperative hemoglobin (Hb), preoperative catheterization, preoperative and postoperative peak flow Qmax, preoperative and postoperative International Prostate Symptom Score (IPSS), and quality of life (QoL)), operating time, postoperative time of trial without catheter (TWOC), hospitalization time, complications classified according to the Clavien–Dindo score (CDS), blood transfusions, and follow-up (FU) period.

### 2.5. Statistical Analysis

For the data analysis, the characteristic and functional values of the individual papers were presented descriptively by calculating means and standard deviation. Since the sample sizes for some surgical techniques were insufficient for further analysis, we refrained from a subdivision into groups at this point. In a meta-analysis, the overall mean of the individual functional values of those surgical techniques that occurred in more than one paper was calculated [16]. As the mean was used in some papers and the median in others, only those reports that employed the mean were taken into account. Not all papers provided information about the standard deviation of preoperative values; therefore, the analysis was limited to postoperative values. To include studies that contained the median and interquartile range, a sensitivity analysis [16] was performed for all studies that reported all complete values (mean and SD, or median, first quartile, and third quartile) for surgical techniques that were employed more than once. For the same techniques, a meta-analysis was performed to calculate the overall proportion [16] of blood transfusions and complications (normal and grade III–V). Due to the large number of variables, presentation by means of forest plots was avoided. Instead, the individual values were presented using boxplots and marking the overall estimate. Pearson’s correlation coefficient was employed to determine the linear relationship between function scores and age, and between the relative frequency of blood transfusions and antiplatelet and anticoagulant medications. The correlation coefficient was calculated across all surgical techniques because the data did not suffice for subgroup analysis. The two-sided significance level of α = 0.05 was used, and all analyses were carried out using the statistical software package R 4.3.2 [17].

## 3. Results

### 3.1. Baseline Characteristics

Baseline characteristics of the patients included in the systematic review and meta-analysis are shown in Table 2.

### 3.2. Functional Outcomes (PVR, Qmax, and IPSS), Length of Stay (LOS) in the Hospital, and Length of Postoperative Catheterization (TWOC, Trial without Catheter)

**Figure 2:**
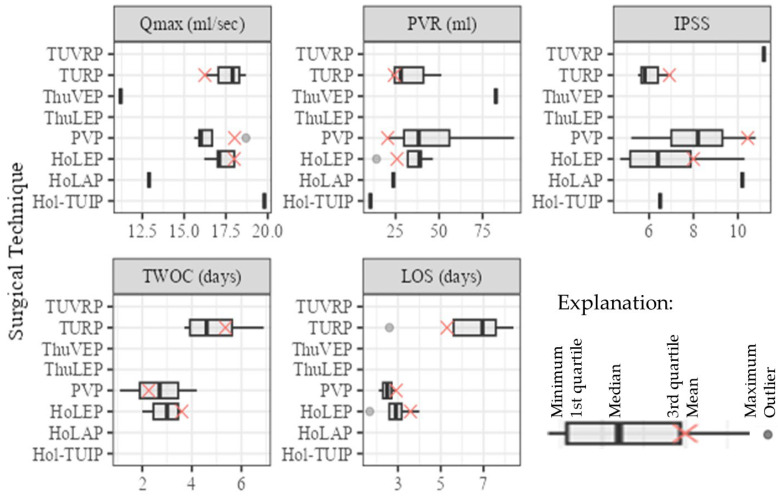
Boxplots of the meta-analysis comparing the surgical techniques in regard to functional outcomes, length of hospital stay, and length of postoperative catheterization (HoLAP—holmium laser ablation of the prostate, HoLEP—holmium laser enucleation of the prostate, Hol-TUIP—holmium laser transurethral incision of the prostate, LOS—length of stay, PVP—photoselective vaporization of the prostate, PVR—post-void residual volume, ThuLEP—thulium laser enucleation of the prostate, ThuVEP—thulium laser vapoenucleation of the prostate, TURP—transurethral resection of the prostate, TUVRP—transurethral vaporization resection of the prostate, TWOC—trial without catheter).

The meta-analysis of postoperative functional data for the respective surgical techniques is shown in Table 3 and Figure 2. The mean follow-up (FU) period differed greatly between the studies and ranged from 1 [18] to 24 [19] months.

For comparison, the postoperative Qmax from the individual studies was 11.2 mL/sec. for ThuVEP [27], 12.9 mL/sec. for HoLAP, and 19.8 mL/sec. for Hol-TUIP [28]. The postoperative IPSS was 11.2 for TUVRP [29], 10.2 for HoLAP [28], and 6.5 for Hol-TUIP [24].

### 3.3. Patient Satisfaction—Preoperative and Postoperative QoL

**Figure 3:**
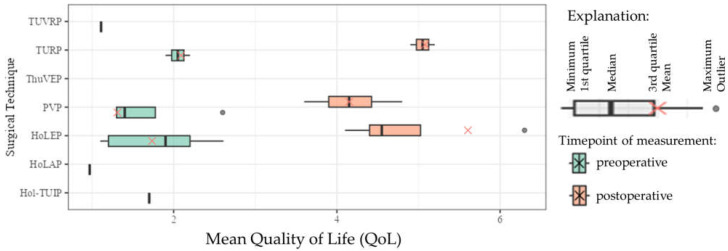
Boxplots of the meta-analysis comparing surgical techniques in regard to mean preoperative and postoperative quality of life (HoLAP—holmium laser ablation of the prostate, HoLEP—holmium laser enucleation of the prostate, Hol-TUIP—holmium laser transurethral incision of the prostate, LOS—length of stay, PVP—photoselective vaporization of the prostate, PVR—post-void residual volume, QoL—quality of life, ThuLEP—thulium laser enucleation of the prostate, ThuVEP—thulium laser vapoenucleation of the prostate, TURP—transurethral resection of the prostate, TUVRP—transurethral vaporization resection of the prostate, TWOC—trial without catheter).

Data concerning preoperative and postoperative QoL are shown in Table 4 and Figure 3. In the figure, the data of the meta-analysis are plotted next to those of the individual studies for comparison, when means were available. The mean follow-up (FU) period differed greatly between the studies and ranged from 1 [18] to 24 [19] months.

For comparison, the postoperative QoL from one selected study was 1.1 for TUVRP [29], 1.0 for HoLAP, and 1.7 for Hol-TUIP [28].

### 3.4. Perioperative and Postoperative Patient Safety, Complications, and Blood Transfusions

A positive linear relationship (r = 0.5007) was observed between the use of anticoagulants or antiplatelet drugs and postoperative transfusions across all surgical techniques [19,22,23,31,32,33,34,35].

**Figure 4:**
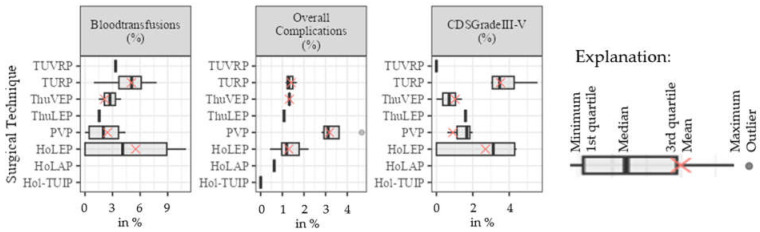
Boxplots of the meta-analysis comparing the different surgical techniques in regard to mean blood transfusions, overall complications, severe complications, and Clavien–Dindo score III-IV as percentages (CDS—Clavien–Dindo score, HoLAP—holmium laser ablation of the prostate, HoLEP—holmium laser enucleation of the prostate, Hol-TUIP—holmium laser transurethral incision of the prostate, PVP—photoselective vaporization of the prostate, ThuLEP—thulium laser enucleation of the prostate, ThuVEP—thulium laser vapoenucleation of the prostate, TURP—transurethral resection of the prostate, TUVRP—transurethral vaporization resection of the prostate).

The rates of overall complications, severe complications (CDS III–IV), and blood transfusions from the meta-analysis are shown in Table 5 and Figure 4. In the figure, the data of the meta-analysis are plotted next to those of individual studies for comparison when means were available.

Not all included publications mentioned the kind of severe complications that occurred in their populations. Transurethral coagulation or bladder clot removal, myocardial infarction, testicular abscess requiring surgery, urethral or bladder neck stricture, aspiration pneumonia, and ureteral ostium injury were mentioned as serious complications. Only a few publications mentioned the number of bladder neck or urethral strictures or the continence rates after surgical interventions. Due to this, no meta-analysis was possible concerning these data.

For comparison, the rates of blood transfusion in the individual studies were 3.3% for TUVRP [29] and 1.55% for ThuLEP [33]. Rates of overall complications were 10.8% for ThuLEP [33], 6.25% for HoLAP, and 0% for Hol-TUIP [28]. Rates of severe complications (CDS III-IV) were 0% for ThuVRP [29] and 1.6% for ThuLEP [33].

### 3.5. Pearson’s Correlation Coefficient (r) between Patient Age and Selected Perioperative and Postoperative Functional and Clinical Parameters

The age of the patients was correlated with clinical and functional parameters (PPC, Table 6). A positive linear correlation was noted between age and inferior outcome on the one hand (higher PVR (r = 0.51) and IPSS (r = 0.42) and higher rates of overall complications (r = 0.54) and blood transfusions (r = 0.45) on the other. Since too few data were available for individual surgical techniques, all surgical techniques were evaluated together here.

## 4. Discussion

In our study, for VOPs, the postoperative Qmax ranged between 16.24 mL/sec. (TURP, transurethral resection of the prostate) and 18 mL/sec. (HoLEP, holmium laser enucleation of the prostate). Values between 24.1 mL/sec. and 28 mL/sec. have been reported in the published literature [39,40]. In a direct comparison, Bertolo et al. [31] found that those <75 years had a significantly better Qmax than those >75 years after thulium laser vapoenucleation of the prostate (ThuVEP). The same was reported by Gild et al. in a comparison of patients <60 years and those >80 years after HoLEP [38]. One of the reasons for the poorer functional outcome in VOPs may be physiological detrusor underactivity in the elderly [41].

A publication by the American Geriatrics Society in 2022 showed that the proportion of persons ≥ 80 years on oral anticoagulation (OAC) increased from 32.4% in 2011 to 43.6% in 2019 [42]. In a report published by Rühle et al. in 2019, patients under OAC who underwent bipolar transurethral resection of the prostate (TURP) required longer bladder irrigation, longer transurethral catheterization, and longer hospital stays compared to the control group. Furthermore, the former patients were more likely to suffer from postoperative urinary retention [43]. As expected, in our group of VOPs, the necessity of a blood transfusion as an expression of a bleeding complication was greater among those under anticoagulation or antiplatelet medication (PCC 0.5007). The European Association of Urology (EAU) guidelines, entitled Management of Non-neurogenic Male LUTSs [12], recommend the use of laser vaporization or enucleation of the prostate for the surgical treatment of BPO in cases of continued use of anticoagulants or antiplatelet drugs. In our study, the percentage of patients on anticoagulation or antiplatelet medication was high for some laser techniques; 40% of patients after HoLEP and 41% of patients after photoselective vaporization of the prostate (PVP) were taking such medication. The use of antiplatelet drugs and/or anticoagulants in the entire collective was between 19% (HoLAP, holmium laser ablation of the prostate) and 41 ± 13% (PVP).

Age, comorbidities, and polypharmacy make VOPs a sensitive population. According to a report published by Yonou et al. in 1999, within a mean follow-up period of 26 months after TURP, 4/13 patients had already died [44]. Some surgical techniques appear to be more appropriate than others depending on the patient’s condition. In 2022, Burtt et al. performed a meta-analysis of various endoscopic techniques for BPO in high-risk patients (defined as “large prostates ≥80 cc and/or taking antithrombotic agents and/or urinary retention and/or age >80 years and/or significant comorbidity”). Compared to TURP, laser techniques such as HoLEP, PVP, and thulium laser were associated with fewer bleeding complications, shorter LOS, and fewer re-interventions, while functional improvement was similar across all surgical techniques [11].

In our study, complication rates for VOPs ranged from 12.97% (HoLEP) to 31.80% (PVP), with severe complications (CDS III-IV) ranging from 0.91% (PVP) to 3.52% (TURP). The complication rates in our study were somewhat higher than those known from the published literature for all age groups. Riedinger et al. [45] reported a complication rate of 9% for TURP compared to 14.5% registered in our study. Yet other authors come to a different conclusion: Bertolo et al. found no difference in complication rates between those <75 years and those >75 years after ThuVEP [31]. However, the rate of severe complications (CDS III-IV) was a maximum of 3.52% for TURP. In other words, all of the studied techniques are largely safe for VOPs.

In our investigation, the LOS for TURP was longer than that for PVP or HoLEP. This confirms the data reported in the published literature. In a meta-analysis published in 2021, Castellani et al. registered a significantly longer TWOC (*p* < 0.00001) and LOS (*p* < 0.00001) for TURP than for PVP [46]. The same was true of HoLEP versus TURP with regard to TWOC and LOS [47,48]. However, a prolonged LOS or later TWOC may pose a risk for VOPs; the risk of venous thromboembolism, pulmonary embolism [49,50,51], pneumonia, and catheter-associated urinary tract infections (CAUTIs) [52] increases with each day.

Very old patients, or VOPs, constitute a heterogeneous group. Selecting those who will benefit from a surgical intervention for BPO is a challenge. A geriatric assessment helps to determine the physical and mental resources of these patients. The importance of a preoperative geriatric assessment in uro-oncological patients was highlighted by Zangl et al. in 2021 [53]. Yet, it is not standard practice prior to BPO surgery. The EAU guidelines on the Management of Non-neurogenic Male LUTSs [12] remain very general in this respect: “The choice of surgical technique depends on […] the patient’s concomitant diseases and whether he is sufficiently fit for anesthesia, as well as on the patient’s preferences, and the willingness to accept surgery-related specific side effects” [12]. However, data on this subject remain ambiguous. Eredics et al., in 2020, registered no difference in intraoperative and perioperative complications, TWOC, and LOS between fit and frail patients [26]. However, Labban et al. found the five-item Frailty Index, a geriatric assessment tool, to be associated with all types of complications (OR 1.50), LOS (OR 1.31), and readmission rates (OR 1.65) after different types of endoscopic BPO surgeries [54].

Although surgical outcomes are presumably worse in VOPs (when compared with the literature), the outcomes are sufficient to recommend surgery in VOPs. The patients’ priorities are surgical safety and QoL. We found that all the examined surgical methods lead to good micturition and associated QoL, especially in comparison with preoperative QoL. The latter ranged from 4.15 to 5.61, which denotes a level of satisfaction between “mostly dissatisfied [QoL = 4]” and “terrible [QoL = 6]”. Postoperatively, the mean QoL score in our study decreased to 1.31 (PVP), 1.74 (HoLEP), and 2.09 (TURP). This translates into a value between pleased [QoL = 1] and close to mostly satisfied [QoL = 2]. Other research groups have achieved similar results. In 2019, Moiroud et al. investigated 305 patients from different age groups after PVP and registered a higher QoL score in patients older than 80 years compared to younger age groups (*p* = 0.04). However, the mean postoperative QoL score in VOPs was 1.5 ± 1.1 [25]. In 2019, Castellani et al. found no difference between the QoL of patients under 75 and over 75 years at 1 (*p* = 0.4) and 12 months (*p* = 0.2) after thulium laser enucleation of the prostate (ThuLEP). Even after HoLEP, Anan et al. registered no difference between those younger and older than 75 years of age in nearly all life domains assessed on the King’s Health Questionnaire at 1, 3, and 6 months [21]. In other words, both younger and older patients may benefit equally from surgery. In addition, QoL may improve further over a period of time after surgical BPO therapy. According to Castellani et al., the quality of life of patients over 75 years of age who underwent ThuLEP improved continuously over a 12-month follow-up period (Δ −3.0 after 1 month, to −3.4 after 6 months, and −3.8 after 12 months) [33]. It must be said that the data were very heterogeneous in our meta-analysis concerning follow-up period as it ranged from 1 to 42 months.

Unfortunately, at the time of the meta-analysis, no data were available for newer interventions such as Rezum™ Water Vapor Therapy or Aquablation^®^ of the prostate that met the inclusion criteria. The same applies for enucleation techniques such as bipolar or diode laser enucleation of the prostate beforehand. This will certainly be an interesting topic in the future. Furthermore, we are missing studies comparing outcomes between young patients vs. VOPs and comparing surgical techniques between VOPs.

The alternative to the surgical treatment of BPO is usually permanent urinary catheterization, which is frequently associated with poor QoL. Ndomba et al. examined the QoL of 202 patients with IUC using the WHOQOL-BREF tool. The median age of the patients was 69 years, and they reported poor QoL in nearly all areas of life [55]. Not only QoL suffers due to IUC; one of the most common complications of IUC is catheter-associated urinary tract infections (CAUTIs). The rate of CAUTIs increases with each day of catheterization [52]. Some authors have even found a correlation between IUC and increased mortality in frail patients [56].

## 5. Conclusions

Patients over 75 years of age experience poorer outcomes and more numerous complications such as blood transfusions after endoscopic treatment for BPO than younger patients. Nevertheless, quality of life (QoL) is noticeably improved by the relevant procedures in the elderly. Currently, it is still left to the clinical judgment and intuition of the treating physician, in conjunction with the patient’s preferences and pre-existing conditions, to decide whether or not to submit a VOP to surgical therapy. Selecting suitable patients for surgery in the presence of a benign disease such as BPO remains a challenge. Tools such as geriatric assessments deserve further investigation in this setting. 

## Figures and Tables

**Table 1:** Cochrane’s collaboration tool for assessing risk of bias (LR = low risk; HR = high risk; UR = uncertain risk).

	Random Sequence Generation (Selection Bias)	Allocation Concealment (Selection Bias)	Blinding of Participants and Personnel (Performance Bias)	Blinding of Outcome Assessment (Detection Bias)	Incomplete Outcome Data (Attrition Bias)	Selective Reporting (Reporting Bias)	Other Bias
Fuschi et al., 2022 [15]	LR	LR	HR	HR	LR	LR	LR

**Table 2:** Baseline characteristics of patients included in systematic review and meta-analysis (ASA = American Society of Anesthesiologists Classification, cc = cubic centimeter, CDS = Clavien–Dindo score, FU = follow-up, Hemoglobin = Hb, HoLAP = holmium laser ablation of the prostate, HoLEP = holmium laser enucleation of the prostate, Hol-TUIP = holmium laser transurethral incision of the prostate, LOS = length of stay, IPSS = International Prostate Symptom Score, PSA = prostate-specific antigen, PVP = photoselective vaporization of the prostate, Qmax = peak flow rates, QoL = quality of life, SD = standard deviation, ThuLEP = thulium laser enucleation of the prostate, ThuVEP = thulium laser vapoenucleation of the prostate, TURP = transurethral resection of the prostate, TUVRP = transurethral vaporization resection of the prostate, TWOC = trial without catheter).

	HoLAP	HoLEP	Hol-TUIP	PVP	TURP	TUVRP	ThuLEP	ThuVEP
n Studies included	1	8	1	7	6	1	1	3
Basic patient characteristics								
Age in years	84.2 (NA)	81.8 (2.6)	84.3 (NA)	82.9 (2.7)	81.3 (3.2)	87.9 (NA)	79 (NA)	79.9 (2.5)
ASA score		2.5 (0.7)		2.7 (NA)	3.2 (NA)	2.5 (NA)		
ASA III–IV in %	62	69 (11)	62	56 (8)	37 (16)	50 (NA)	57 (NA)	47 (11)
Antiplatelet drugs and/or anticoagulants in %	19 (NA)	40 (10)	23 (NA)	41 (13)	32 (28)		20 (NA)	27 (7)
Preoperative prostate volume in cc	32.9 (NA)	92.6 (19.7)	32.4 (NA)	61.9 (12.8)	60.5 (9.1)	61.6 (NA)	64 (NA)	57.4 (11.3)
Preoperative Catheterization in %		42 (13)		44 (17)	56 (36)		30 (NA)	27 (0)
Preoperative laboratory and functional values								
PSA in ng/mL	13.1 (NA)	7.4 (4.4)	4.8 (NA)	5.72 (4.6)	2.6 (NA)		2.9 (NA)	4.5 (3.4)
Hb in g/dL		12.6 (1.6)		14.1 (0.1)	13.8 (0.5)	12.3 (NA)		13.9 (NA)
PVR in mL		147.8 (91.1)		171.5 (43.9)	87 (NA)		55 (NA)	111.9 (16.8)
Qmax in mL/sec.		8.9 (0.6)		6.6 (2.0)	7.7 (0.8)		7.8 (NA)	8.4 (0.8)
IPSS		19.5 (2)		21.8 (1.8)	23.4 (3.6)		26 (NA)	24.1 (1.4)
QoL		4.7 (0.9)		4.1 (0.4)	5.1 (0.2)		4.5 (NA)	4 (NA)
Perioperative parameters and patient safety								
Operating time in min.	54 (NA)	86.9 (8.7)	31.3 (NA)	53.4 (17.5)	61.7 (7.8)	40.0 (NA)	55 (NA)	71.2 (15.0)
TWOC in days		2.7 (0.76)		2.5 (1.3)	4.4 (1.8)		2 (NA)	2.5 (0.7)
LOS in days		2.9 (0.8)		2.3 (0.9)	5.7 (2.4)		3 (NA)	3 (0)
Overall complications in %	6 (NA)	13 (7)	0 (NA)	34 (8)	14 (3)		11 (NA)	13 (0)
Complications CDS Grade III–V		2 (2)		1 (1)	4 (1)	0 (NA)	2 (NA)	1 (1)
Hb in g/dL		11.9 (1.2)		13.8 (0.3)	11.9 (NA)			15.4 (NA)
Blood transfusions in %		5 (6)		2 (2)	5 (3)	3 (NA)	2 (NA)	3 (2)
Postoperative laboratory and functional parameters								
PSA in ng/mL		1.4 (0.8)		1.7 (0.3)				4.4 (NA)
PVR in mL.	23.7 (NA)	34.1 (12.4)	10.5 (NA)	47.5 (31.9)	33.5 (12.3)			41.3 (58.4)
Qmax in mL/sec.	12.9 (NA)	17.3 (0.8)	19.8 (NA)	16.9 (1.4)	17.6 (1.3)			13.4 (3.0)
IPSS	10.2 (NA)	6.8 (2.2)	6.5 (NA)	9.2 (3.8)	6.1 (0.8)	11.2 (NA)		6.5 (NA)
QoL	1.0 (NA)	1.8 (0.7)	1.7 (NA)	1.9 (0.8)	2.1 (0.2)	1.1 (NA)		1 (NA)

Note: If not specified, the values are mean ± SD.

**Table 3:** Meta-analysis of functional outcomes, length of stay, and length of postoperative catheterization (CI—confidence interval, HoLEP—holmium laser enucleation of the prostate, IPSS—International Prostate Symptom Score, LOS—length of stay, PVP—photoselective vaporization of the prostate, PVR—post-void residual volume, QoL—quality of life, TURP—transurethral resection of the prostate, TWOC—trial without catheter). Note: The values mentioned in the table are n or means with lower and upper boundaries of the CI in brackets. If the confidence intervals do not overlap, a significant difference between the techniques can be inferred. *p*-value regarding the test of heterogeneity between individual studies: ^+^ *p* > 0.05 (not significant), * *p* < 0.05 (significant). ° = two subgroups (after monopolar and bipolar TURP [20]) from one publication were meta-analyzed here, but they were marked as single cohorts.

	HoLEP	TURP	PVP
Qmax in mL/sec.	18.00 ^+^ (17.95–18.05)	16.24 ^+^ (16.20–16.28)	18.02 (17.30–18.74)
Number of analyzed studies	4 [15,21,22,23]	3 * [15,20]	2 * [24,25]
Patient numbers	217	267	210
PVR in mL.	25.81 ^+^ (23.92–27.71)	24.25 ^+^ (23.66–24.84)	20.43 ^+^ (18.90–21.97)
Number of analyzed studies	4 [15,21,22,23]	4 * [15,20,26]	2 [24,25]
Patient numbers	217	321	210
IPSS	8.00 ^+^ (7.96–8.03)	6.92 ^+^ (6.88–6.95)	10.44 ^+^ (9.99–10.9)
Number of analyzed studies	4 [15,21,22,23]	3 * [15,20]	2 [24,25]
Patient numbers	217	267	210
TWOC in days	3.60 ^+^ (3.55–3.64)	5.36 * (5.30–5.41)	2.26 ^+^ (2.02–2.50)
Number of analyzed studies	4 [15,21,22,23]	4 [15,20,26]	3 [19,24,25]
Patient numbers	217	321	233
LOS in days	3.59 ^+^ (3.51–3.66)	5.30 * (5.22–5.37)	2.92 ^+^ (2.73–3.11)
Number of analyzed studies	4 [15,21,22,23]	4 ° [15,20,26]	3 [19,24,25]
Patient numbers	217	321	233

**Table 4:** Meta-analysis comparing surgical techniques in regard to mean preoperative and postoperative quality of life scores (CI—confidence interval; HoLEP—holmium laser enucleation of the prostate, PVP—photoselective vaporization of the prostate, QoL—quality of life, TURP—transurethral resection of the prostate). Note: The values mentioned in the table are n or means, with lower and upper boundaries of the CI in brackets. If the confidence intervals do not overlap, a significant difference between the techniques can be inferred. *p*-value regarding the test of heterogeneity between individual studies: ^+^ *p* > 0.05 (not significant), * *p* < 0.05 (significant). ° = two subgroups (after monopolar and bipolar TURP [20]) from one publication were meta-analyzed here, but they were marked as single cohorts.

	HoLEP	TURP	PVP
Preoperative QoL	5.61 ^+^ (5.43–5.78)	5.08 * (4.97–5.20)	4.15 ^+^ (4.07–4.24)
Number of analyzed studies	3 [21,22,23]	2 ° [20]	4 [19,24,25,30]
Numbers of patients	121	163	819
Postoperative QoL	1.74 ^+^ (1.52–1.95)	2.09 * (1.98–2.20)	1.31 ^+^ (1.25–1.37)
Number of analyzed studies	3 [21,22,23]	2 ° [20]	2 [24,25]
Numbers of patients	291	267	819

**Table 5:** Meta-analysis of perioperative and postoperative complications and blood transfusions as percentages (CDS—Clavien–Dindo score, HoLEP—holmium laser enucleation of the prostate, PVP—photoselective vaporization of the prostate, TURP—transurethral resection of the prostate, ThuVEP—transurethral vapoenucleation of the prostate). Note: The values mentioned in the table are means, with the lower and upper boundaries of the confidence interval in brackets. If the confidence intervals do not overlap, a significant difference between the techniques can be inferred. *p*-value regarding the test of heterogeneity between individual studies: ^+^ *p* > 0.05 (not significant), * *p* < 0.05 (significant).

	HoLEP	TURP	ThuVEP	PVP
Overall complications	12.97 * (10.39–16.08)	14.15 ^+^ (10.59–18.66)	13.27 ^+^ (9.18–18.81)	31.80 ^+^ (27.84–36.04)
Number of analyzed studies	6 [15,18,22,23,28,36]	3 [15,26,32]	2 [31,32]	4 [25,28,35,37]
Numbers of patients	539	290	194	493
Complications CDS III–IV	2.70 ^+^ (1.45–4.95)	3.52 ^+^ (2.17–5.66)	1.03 ^+^ (0.26–4.03)	0.91 ^+^ (0.34–2.43)
Number of analyzed studies	5 [15,18,22,23,36]	4 [15,26,32,34]	2 [31,32]	3 [25,35,37]
Numbers of patients	368	458	194	429
Blood transfusions	5.52 ^+^ (3.59–8.40)	5.06 (3.39–7.49)	2.14 ^+^ (0.82–5.46)	2.45 ^+^ (1.65–3.64)
Number of analyzed studies	4 [15,22,23,38]	4 [15,26,32,34]	2 [31,32]	4 [19,30,35,37]
Numbers of patients	363	458	194	978

**Table 6:** Pearson’s correlation coefficient (r) between patient age and selected perioperative and postoperative functional and clinical parameters across all surgical techniques (CDS—Clavien–Dindo score, IPSS—International Prostate Symptom Score, LOS—length of stay, PO—postoperative, Qmax—peak flow rates, PVR—post-void residual volume, QoL—quality of life, TWOC—trial without catheter).

PO PVR [15,19,20,21,24,25,26,27]	0.51
PO Qmax [15,19,20,21,24,25,27]	−0.14
PO IPSS [15,19,20,21,25,29,36]	0.42
PO QoL [19,20,21,24,25,36]	−0.43
Blood transfusions [15,19,26,29,30,31,32,33,34,37]	0.45
Overall complications [15,18,25,26,29,31,32,33,34,36,37]	0.54
Complications CDS III-IV [15,18,25,26,28,31,32,33,36,37]	0.12
TWOC [15,19,20,21,24,25,26]	−0.10
LOS [15,19,20,21,24,25,26]	−0.13

## Data Availability

The data presented in this study are available on request from the corresponding author.

## Abbreviations

ASA	American Society of Anesthesiologists Classification
BPH	Benign prostatic hyperplasia
BPO	Benign prostatic obstruction
CAUTIs	Catheter-associated urinary tract infections
cc	Cubic centimeters
CDS	Clavien–Dindo score
FU	Follow-up
Hb	Hemoglobin
HoLAP	Holmium laser ablation of the prostate
HoLEP	Holmium laser enucleation of the prostate
Hol-TUIP	Holmium laser transurethral incision of the prostate
IPSS	International Prostate Symptom Score
IUC	Indwelling urinary catheter
KTP	Potassium titanyl phosphate
LOS	Length of (hospital) stay
LUTSs	Lower urinary tract symptoms
MM	Multimorbidity
OAC	Oral anticoagulation
OECD	Organization for Economic Cooperation and Development
PSA	Prostate-specific antigen
PVP	Photoselective vaporization of the prostate
QoL	Quality of life
r	Pearson’s correlation coefficient
ThuLEP	Thulium laser enucleation of the prostate
TURP	Transurethral resection of the prostate
TUVRP	Transurethral vaporization resection of the prostate
TWOC	Trial without catheter
USA	United States of America
